# Persistent Hyperammonemia and Cerebral Edema Following Gastrostomy Tube Placement in a Post-bariatric Patient: A Diagnostic Dilemma

**DOI:** 10.7759/cureus.90875

**Published:** 2025-08-24

**Authors:** Andy Burk, Abbas Merchant, Haashim Rahman, Aaryan Patel, Ishan Deshmukh, Victor Aisogun, Amy Yu, Zaheer Irani, Shriha Patel, Constantino G Lambroussis

**Affiliations:** 1 Anesthesiology, Albany Medical College, Albany, USA; 2 Anesthesia, Lake Erie College of Osteopathic Medicine, Erie, USA; 3 Anesthesiology, Lake Erie College of Osteopathic Medicine, Erie, USA; 4 Physical Medicine and Rehabilitation, Lake Erie College of Osteopathic Medicine, Erie, USA; 5 Vascular Surgery, Lake Erie College of Osteopathic Medicine, Elmira, USA; 6 Anesthesiology and Critical Care, University of California San Diego, San Diego, USA; 7 Radiology, Rochester General Hospital, Lake Erie College of Osteopathic Medicine, Rochester, USA; 8 Psychiatry, Lake Erie College of Osteopathic Medicine, Elmira, USA; 9 Osteopathic Medicine / Family Medicine, Lake Erie College of Osteopathic Medicine, Elmira, USA

**Keywords:** gastric tube complication, global cerebral edema, g-tube, hyperammonemia-encephalopathy, metabolic encephalopathy, neurocritical care, postoperative complicaiton, roux-en-y gastric bypass (rygb), sepsis

## Abstract

A 45-year-old woman with a history of Roux-en-Y gastric bypass (RYGB) and recent cholangitis presented with acute encephalopathy, seizures, and concern for anoxic brain injury. She was intubated for acute respiratory failure and found to have striking hyperammonemia with serum ammonia levels exceeding 300 µmol/L, in the absence of overt liver dysfunction. Her clinical course was complicated by septic shock, multifocal pneumonia, and gastrostomy tube (G-tube) malfunction. Despite aggressive medical management, including broad-spectrum antibiotics and lactulose, ammonia levels remained persistently elevated. CT and MRI of the brain revealed cerebral edema and diffuse cortical diffusion abnormalities concerning for metabolic encephalopathy or anoxic injury. Continuous renal replacement therapy (CRRT) was initiated due to worsening encephalopathy and acute kidney injury (AKI). Notably, imaging demonstrated malposition of the G-tube with distal placement in the excluded gastric remnant; an anatomic reservoir bypassed in Roux-en-Y that is not exposed to normal enteric flow. G-tube revision and eventual conversion to gastrojejunostomy tube (GJ-tube) led to a rapid decline in ammonia levels and improvement in neurologic status. We present a severe and unusual case of non-hepatic hyperammonemia, likely precipitated by altered post-bariatric anatomy and nitrogenous substrate accumulation in the excluded gastric remnant. While hyperammonemia is a well-described complication of liver failure and urea cycle disorders, it is rarely attributed to anatomical disruption in gastrointestinal transit. This report underscores the importance of considering altered post-surgical anatomy in the differential for unexplained metabolic encephalopathy and supports early imaging of enteral access in patients with Roux-en-Y anatomy and neurologic decline. Timely recognition and intervention may prevent irreversible neurologic injury and reduce the need for prolonged renal support.

## Introduction

Hyperammonemia, characterized by elevated plasma ammonia levels, is a potentially life-threatening condition that can lead to cerebral edema, seizures, and coma. It is classically associated with hepatic dysfunction due to impaired urea cycle clearance. Non-hepatic hyperammonemia (NHH) is an increasingly recognized and underdiagnosed condition, seen in settings such as urea-splitting bacterial infections, renal dysfunction, inborn errors of metabolism, and altered gastrointestinal anatomy [1,2].

Among surgical populations, patients with a history of the Roux-en-Y gastric bypass (RYGB) present unique metabolic challenges. In the RYGB, a small proximal pouch is created from the lesser curvature of the stomach, excluding the fundus, body, and antrum. The excluded gastric remnant is bypassed but remains anatomically intact and continues to secrete gastric contents, creating a potential site for stasis, bacterial overgrowth, and metabolic complications [3,4]. While RYGB is not routinely associated with hyperammonemia, there have been isolated reports suggesting that stagnant proteinaceous material in the excluded stomach or small bowel, especially in the presence of urease-producing organisms, may contribute to significant ammonia accumulation in the absence of liver disease [5].

We present a case of a critically ill 45-year-old woman with persistent and severe non-hepatic hyperammonemia, ultimately requiring continuous renal replacement therapy (CRRT). Her clinical course was complicated by toxic-metabolic encephalopathy and radiographic evidence of cerebral edema. Notably, she had undergone a transgastric ERCP via percutaneous gastrostomy tube (G-tube), and subsequent malfunction of that drainage tube may have contributed to the accumulation of nitrogenous substrates in the excluded stomach. This case underscores a rare but clinically significant mechanism of hyperammonemia in patients with surgically altered gastrointestinal anatomy and emphasizes the importance of considering anatomical, infectious, and procedural factors when evaluating NHH in the intensive care unit (ICU) setting.

## Case presentation

A 45-year-old woman with a complex medical and surgical history, including hypertension, chronic migraines, opioid use disorder controlled with buprenorphine, and RYGB with subsequent G-tube placement for biliary access, was admitted with acute encephalopathy. Prior to admission, her baseline mental status was intact. Over the following 48 hours, she developed progressive confusion, facial twitching, and unresponsiveness, which then prompted intubation for airway protection. Workup revealed Group B *Streptococcus bacteremia*, presumed to be secondary to a chronically leaking G-tube site, with features of associated septic shock and acute hypoxemic respiratory failure.

Initial labs (Table 1) revealed elevated white blood cell count of 33.7 ×10³/μL (reference range 4-11 ×10³/μL), a mild transaminitis with an aspartate aminotransferase (AST) level of 85 U/L and an alanine aminotransferase (ALT) level of 40 U/L (reference range AST: 5-45, ALT: 7-56, total bilirubin 3.5 mg/dL (reference range 0.1 to 1.2 mg/dL), and creatinine increased from a baseline of 0.5 to 1.56 mg/dL. Arterial blood gas showed alkalemia (pH 7.56) with mild hypoxia. Critically, serum ammonia was elevated to 301 μmol/L (reference ≤32 μmol/L), raising concern for hyperammonemic encephalopathy.

**Table 1:** Initial laboratory values WBC: white blood cell, AST: aspartate transaminase, ALT: alanine transaminase

Parameter	Value	Reference range
WBC	33.7 ×10³/μL	4-11 ×10³/μL
AST	85 U/L	5-45 U/L
ALT	40 U/L	7-56 U/L
Total bilirubin	3.5 mg/dL	0.1- 1.2 mg/dL
Creatinine	1.56 mg/dL	0.62- 1.1 mg/dL
Serum pH	7.56	7.35-7.45
Serum ammonia	301 μmol/L	≤32 μmol/L

Urine and gastrostomy site cultures grew *Enterococcus faecium*, *Proteus mirabilis*, *Klebsiella pneumoniae*, and *Candida albicans*, suggesting polymicrobial colonization or infection of the G-tube tract. These findings likely reflect chronic contamination of the tube in the outpatient setting, as the patient had been discharged home after G-tube placement and required multiple exchanges for leakage and displacement. Despite broad-spectrum antibiotics and antifungals, ammonia levels remained critically elevated. Interventional radiology confirmed persistent leakage and malpositioning despite upsizing and fluoroscopic repositioning, limiting safe enteral use.

**Figure 1:**
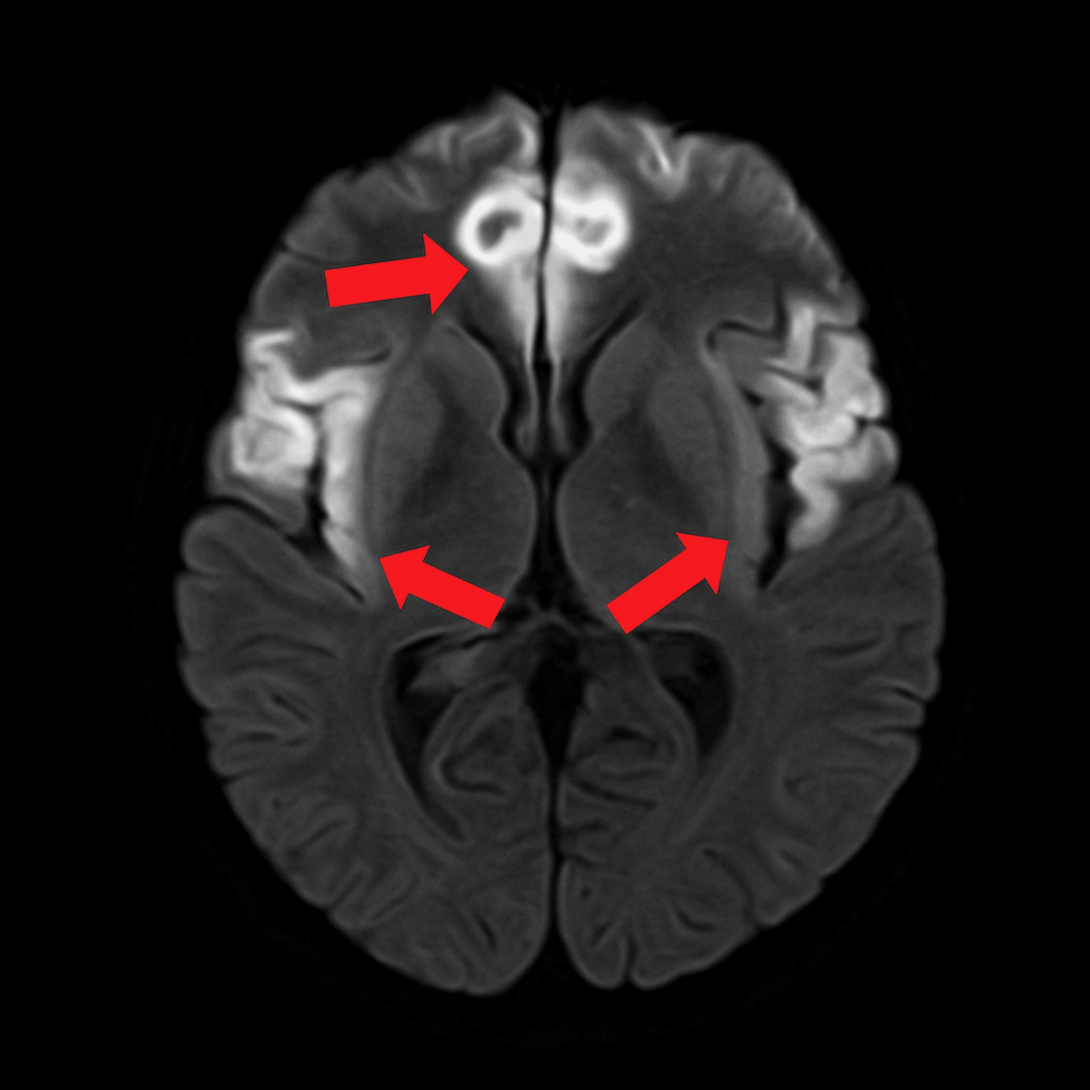
Axial DWI MRI of the brain Cerebral cortical restricted diffusion, as denoted by the red arrows, is indicated by the hyperintense and curvilinear regions most prominent in close proximity to the paramedian frontal lobes and subcortical regions, consistent with toxic-metabolic or anoxic injury. DWI: diffusion-weighted imaging, MRI: magnetic resonance imaging

Due to refractory hyperammonemia and worsening neurologic status, continuous renal replacement therapy (CRRT) was initiated, resulting in gradual ammonia clearance and clinical improvement. The liver function of the patient remained stable, underscoring a non-hepatic etiology. An MRI of the brain showed diffuse cortical and subcortical restricted diffusion, consistent with toxic-metabolic or anoxic injury, as shown in Figure 1. The ophthalmology service was consulted, which noted trace bilateral optic nerve head edema without frank papilledema. The neurology service favored the diagnosis of nonhepatic hyperammonemic encephalopathy, with seizure activity contributing to the overall encephalopathic status.

**Figure 2:**
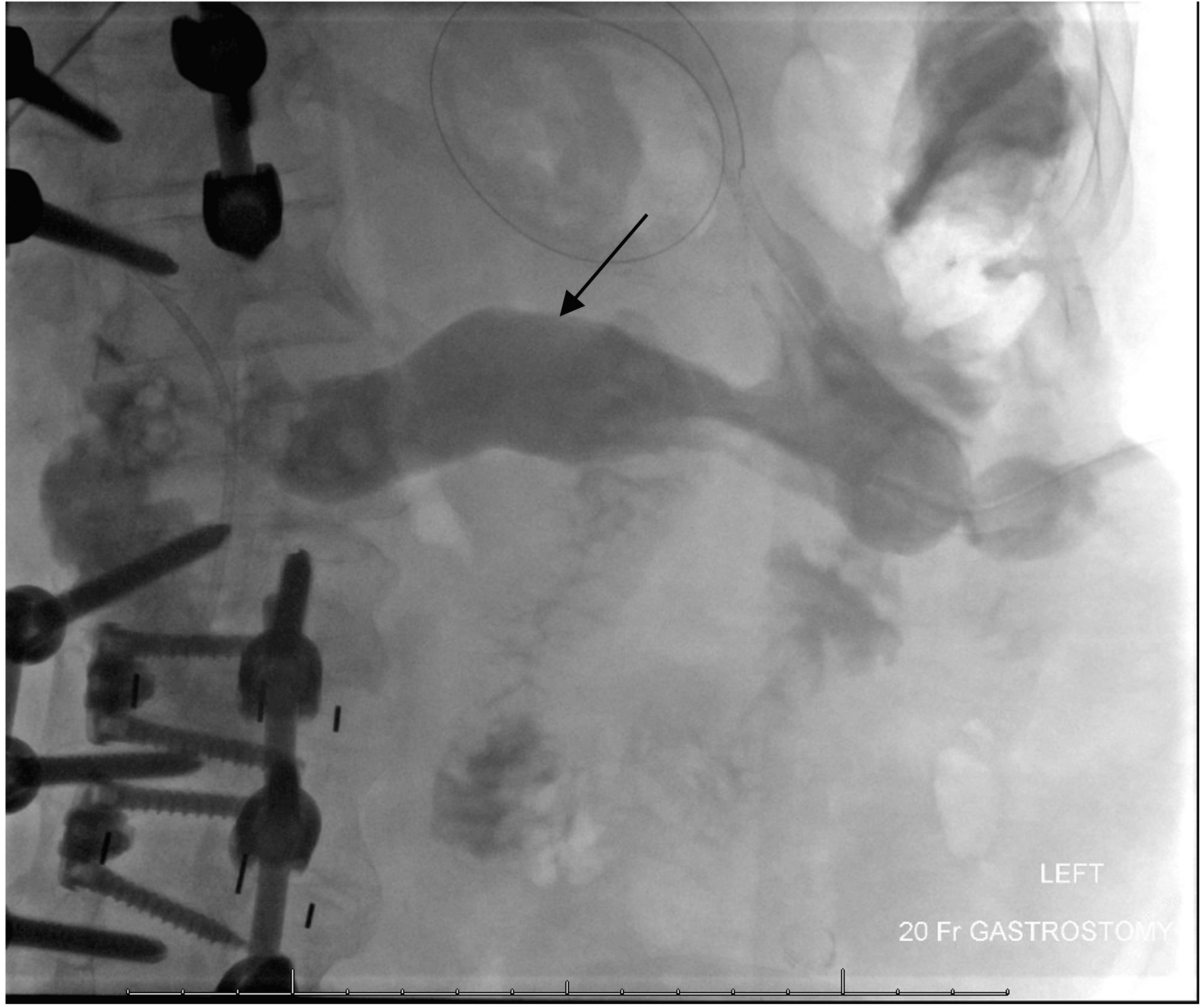
Fluoroscopic image from a gastrostomy tube contrast study Fluoroscopic image from a gastrostomy tube contrast study demonstrating pooling of contrast (arrow) within the excluded gastric remnant in a patient with Roux-en-Y gastric bypass anatomy. This finding raised concern for stasis of nitrogenous material and bacterial overgrowth as a potential source of persistent hyperammonemia.

Nephrology escalated care to continuous venovenous hemodiafiltration (CVVH) using Phoxilium at 30 mL/kg/hr, avoiding intermittent dialysis due to osmotic shift risk. Ammonia levels rebounded with the reintroduction of enteral feeds. Notably, the G-tube had been placed during a transgastric ERCP into the excluded stomach, a segment isolated from the alimentary tract post-RYGB. Serial fluoroscopy confirmed tube placement in the gastric remnant but demonstrated contrast stasis, raising concern for stagnant nitrogenous substrate accumulation. Figure 2 shows fluoroscopic imaging with the contrast stasis in our patient.

Despite ongoing CRRT and maximal medical therapy, the ammonia remained intermittently elevated. Surgical and gastroenterology teams hypothesized that bacterial overgrowth and urease activity in the excluded stomach contributed to excess ammonia production. Thus, feeds were paused, and GJ conversion was considered.

**Figure 3:**
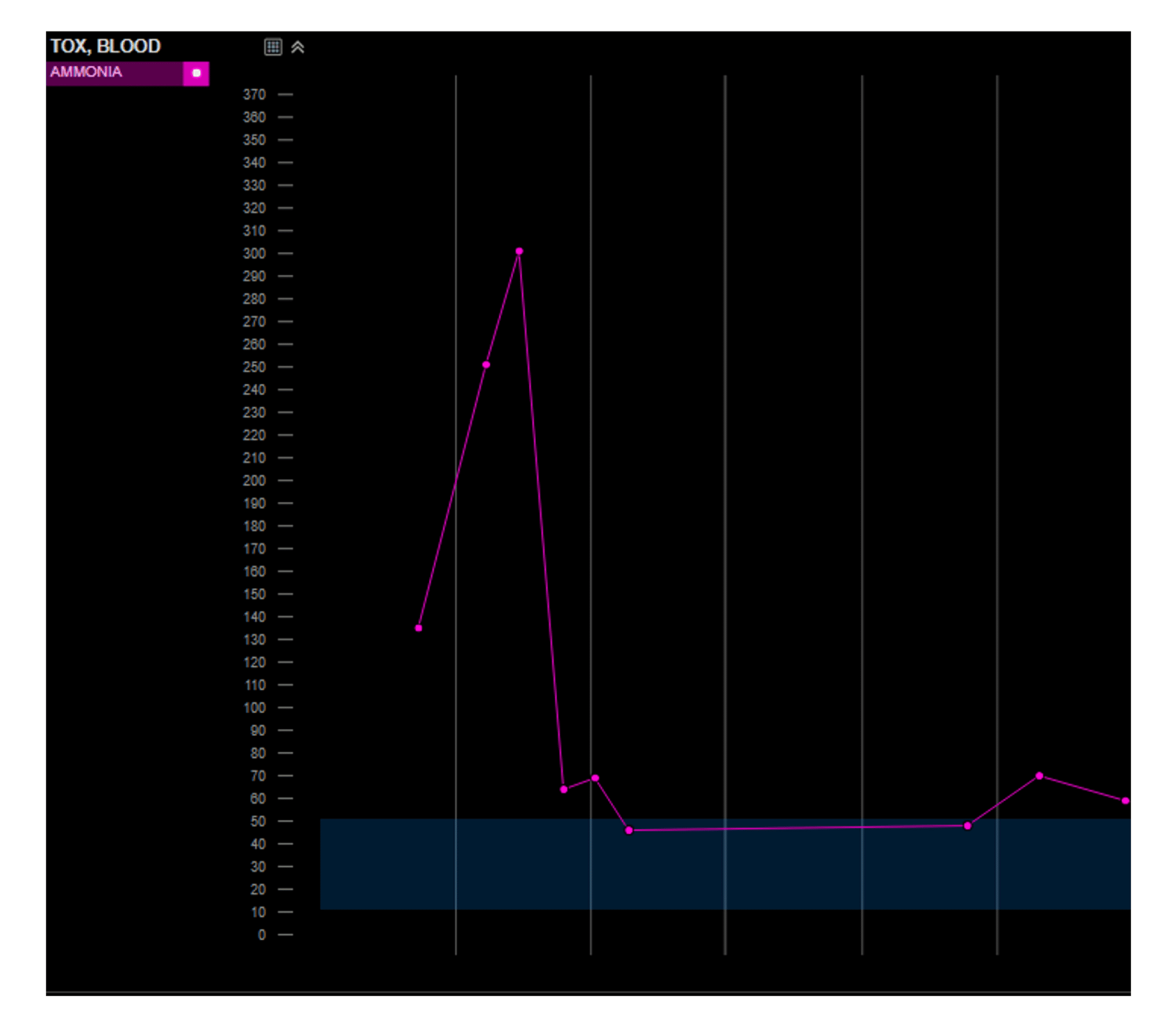
Serum ammonia trend during hospitalization Serum ammonia trend during hospitalization. Peak level exceeded 300 µmol/L, followed by a sharp decline after initiation of continuous renal replacement therapy (CRRT) and bowel rest. Transient elevation occurred with the reintroduction of enteral feeds, supporting an enteric source of ammonia. Subsequent stabilization was achieved after further G-tube management.

Over the next several days, ammonia levels declined sharply and remained low with continued CRRT and bowel rest. Neurologic status gradually improved, and she was weaned off CRRT and extubated. The patient remained amnestic to her ICU course but was alert and interactive by day 30 and discharged to long-term acute care for rehabilitation. Figure 3 shows the serum ammonia trend of our patient during their hospitalization. A GJ conversion was considered to bypass the excluded gastric remnant, though clinical improvement was observed with bowel rest and CRRT prior to the procedure being performed.

## Discussion

Nonhepatic hyperammonemia (NHH) is an underrecognized but potentially life-threatening condition that can rapidly progress to cerebral edema and metabolic encephalopathy. While typically associated with liver failure or inborn errors of metabolism, NHH may also arise in critical illness, malignancy, chemotherapy, gastrointestinal bleeding, urinary tract infections, and post-surgical alterations of gastrointestinal anatomy [1-3]. In this case, the patient’s persistent hyperammonemia in the absence of liver dysfunction, combined with her altered gastrointestinal anatomy and evidence of cerebral injury, presented a diagnostic and management challenge.

Our patient’s RYGB excluded a large portion of the stomach and biliopancreatic limb from the alimentary stream. This creates a nutrient-poor, acid-deficient segment prone to bacterial overgrowth [4,5]. Placement of a percutaneous G-tube into the excluded stomach, as was done in this patient for biliary access, may result in the accumulation of nitrogenous substrates and promote urease-producing microbial colonization. These microbes, particularly urease-producing organisms, hydrolyze urea and amino acids such as glutamine into ammonia, leading to systemic accumulation in the absence of hepatic clearance [6,7]. Recurrent leakage and dislodgement of the G-tube likely worsened this effect by allowing extraluminal seepage and reducing clearance of the gastric remnant’s contents.

This presentation was distinct from classic hepatic encephalopathy. The patient had no prior history of liver disease, and her synthetic liver function remained intact, with normal international normalized ratio (INR) and serum albumin levels. Transaminase elevations were mild and non-specific. Despite these reassuring hepatic markers, her serum ammonia was markedly elevated (>300 µmol/L) and refractory to standard therapies including lactulose and rifaximin. Neuroimaging revealed diffuse cortical and subcortical diffusion restriction, consistent with cytotoxic edema, a hallmark of acute hyperammonemic encephalopathy rather than the chronic changes typically associated with liver-related encephalopathy. Given the significant risk of cerebral herniation, CRRT was initiated early and effectively reduced serum ammonia levels while minimizing the hemodynamic and osmotic fluctuations associated with intermittent hemodialysis [8].

The etiology of hyperammonemia in the setting of gastrointestinal surgery is underrecognized. Prior case reports describe similar phenomena in patients with blind loop syndrome, jejunoileal bypass, or RYGB with enteric fistulae, where ammonia is produced in excess by bacterial fermentation [6]. Recurrent hyperammonemia following reintroduction of enteral nutrition, despite ongoing CRRT, further implicated the excluded gastric remnant as a persistent ammonia source, likely due to ongoing bacterial fermentation of nitrogenous substrates. This highlights the importance of considering G-tube position, function, and target anatomy in post-bariatric patients with unexplained encephalopathy. We recommend the consideration of G-tube malposition in post-bariatric surgical patients who subsequently develop encephalopathy, and recommend the early monitoring of serum ammonia levels in this patient population.

This case also underscores the utility of a multidisciplinary approach. Nephrology, neurology, interventional radiology, gastroenterology, and surgery each contributed to diagnostic clarification and risk mitigation. Ophthalmology's identification of optic disc edema suggested early intracranial hypertension. IR’s confirmation of the G-tube’s location and function informed the decision to transition to bowel rest and ultimately consider GJ conversion. The patient's recovery supports early recognition and aggressive intervention in NHH, even in the absence of liver dysfunction.

## Conclusions

This case highlights a rare but serious complication of post-bariatric surgical anatomy: nonhepatic hyperammonemia, likely driven by stagnant nitrogenous substrates and bacterial overgrowth within the excluded gastric remnant. In patients with RYGB and recent G-tube placement into the bypassed stomach, persistent hyperammonemia should prompt early consideration of an enteric source, even in the absence of liver dysfunction. Timely recognition of this phenomenon is critical, as delays in diagnosis may lead to cerebral edema, seizures, and irreversible neurologic injury. A high index of suspicion, prompt neuroimaging, and early initiation of renal replacement therapy were central to the favorable outcome in this case. Multidisciplinary coordination and a clear understanding of altered gastrointestinal anatomy and enteric stasis are essential in managing hyperammonemic encephalopathy in complex surgical patients.
